# A Data Mining-Based Analysis of Core Herbs on Different Patterns (*Zheng*) of Non-Small Cell Lung Cancer

**DOI:** 10.1155/2021/3621677

**Published:** 2021-06-28

**Authors:** Xiangjun Qi, Zehuai Guo, Qianying Chen, Wanning Lan, Zhuangzhong Chen, Wenmin Chen, Lizhu Lin

**Affiliations:** ^1^The First Clinical School of Guangzhou University of Chinese Medicine, Guangzhou, China; ^2^The First Affiliated Hospital of Guangzhou University of Chinese Medicine, Guangzhou, China; ^3^Cancer Project Team of China Center for Evidence Based Traditional Chinese Medicine, Beijing, China

## Abstract

**Objective:**

To explore the role of Chinese prescriptions in non-small cell lung cancer (NSCLC) and provide references for the application of herbs and prescriptions.

**Methods:**

Randomized and quasirandomized controlled clinical trials on Chinese herbal medicine in the treatment of NSCLC were collected from seven databases to establish a database of prescriptions on NSCLC. Data-mining analyses were performed by RStudio (v4.0.3) software.

**Results:**

A total of 970 prescriptions were obtained from 945 included studies, involving 7 syndromes and 428 herbs. The main patterns of NSCLC included *qi* deficiency pattern, *yin* deficiency pattern, blood deficiency pattern, kidney deficiency pattern, heat toxin pattern, phlegm-dampness pattern, and blood stasis pattern. High-frequency herbs on NSCLC were Astragali Radix (Huangqi), Atractylodis Macrocephalae Rhizome (Baizhu), Glycyrrhizae Radix Rhizome (Gancao), Poria (Fuling), Ophiopogonis Radix (Maidong), Hedyotidis Diffusae Herba (Baihuasheshecao), Codonopsis Radix (Dangshen), and Glehniae Radix (Beishashen). The properties of the herbs were mainly cold, warm, and mild. The flavors of the herbs were mainly sweet, bitter, and pungent. The main meridian tropisms were Lung Meridian of Hand-Taiyin, Spleen Meridian of Foot-Taiyin, and Stomach Meridian of Foot-Yangming.

**Conclusion:**

Applying clearing and tonifying method by targeting the lung and spleen was the most frequently used therapy in the treatment of NSCLC. This study offered a glimpse of unique views of traditional Chinese medicine on NSCLC and may benefit the treatment of NSCLC.

## 1. Introduction

Lung cancer is a major cause of deaths worldwide, with approximately 2.2 million new cases and 1.8 million deaths in 2020 [1]. Non-small-cell lung cancer (NSCLC) is the most frequent type of lung cancer, accounting for 80%–85% of total cases [2] and carrying overall 5-year survival rate lower than 15% [3]. Originated from ancient China, traditional Chinese medicine (TCM) is a complementary therapy that has developed rich experience in treating cancers and has been reported to benefit patients with NSCLC [4, 5]. The principle of TCM in practice is syndrome or pattern (*zheng*) differentiation and treatment. A patient may manifest different patterns in different stages of a disease, while patients with different diseases may share the same pattern. Prescription or formula prescribed by qualified TCM practitioners is the main form of TCM in treating diseases. According to TCM theory, proper combinations of herbs can make the best use of therapeutic effects by mutual reinforcement and mutual assistance, while inappropriate combinations of herbs may reduce efficacy, increase toxicity, or cause side effects by mutual restraint, mutual suppression, and mutual inhibition. Thus, TCM practitioners not only prescribe formulae based on diagnosis and syndrome differentiation, but deliberately modify them according to patient's specific condition and changing pattern in the development of a disease. However, it is far from simplicity for TCM practitioners to apply and modify formulae flexibly to achieve favorable effects. To explore basic therapeutic principles, identify the main syndromes and common herbs may help to address concerns about the effective application of TCM.

In recent years, accumulative clinical trials on formulae in treating NSCLC have been conducted. Therefore, we collected formulae on NSCLC from randomized and quasirandomized controlled clinical trials to perform a data mining-based analysis, which may provide an available reference for TCM practitioners in the treatment of NSCLC.

## 2. Materials and Methods

### 2.1. Literature Research

A comprehensive literature research was conducted in seven databases including China National Knowledge Infrastructure (CNKI), Wanfang Database, Chinese Scientific Journal Database (VIP), Sinomed, PubMed, Embase, and Cochrane Library based on the following research terms: “traditional Chinese medicine,” “Chinese herb,” “Chinese drug,” “Chinese formula,” “Chinese prescription,” “non-small cell lung cancer,” “adenocarcinoma of lung,” and “squamous cell lung carcinoma.”

### 2.2. Study Selection

Studies that satisfied the following criteria were included. (1) The type of study was limited to randomized or quasirandomized controlled trial. (2) Participants were pathologically diagnosed with NSCLC. (3) Oral Chinese formula or Chinese patent medicine was used alone or in combination with radiotherapy, chemotherapy, targeted therapy, or immunotherapy in trial group. (4) The composition of oral Chinese formula or Chinese patent medicine was recorded specifically. (5) Outcomes were assessed by objective measurements from laboratory tests or imaging techniques, or by measurements from medical staffs, patients, or other informants. (6) Studies provided information that could be used to identify the pattern (*zheng*).

Studies that met the following criteria were excluded. (1) Studies included participants who were diagnosed with NSCLC and other tumors. (2) Rate of losing follow-up exceeded 20%.

### 2.3. Data Extraction and Normalization

Two researchers (XJQ and ZHG) independently searched the databases and screened relevant literature. Any disagreement would be discussed or a further decision would be made by QYC. For each article, the title, author, publication date, diagnostic procedure, the composition of prescription, and *zheng*'s related information were recorded. Duplicate prescriptions were recorded only once. *People's Republic of China Pharmacopoeia* (2015 Edition) [6] was referred to standardize names and characteristics of herbs and *World Health Organization (WHO) Evidence-Based Complementary and Alternative Medicine International Standard Terminologies on Traditional Medicine in the Western Pacific Region* [7] was referred to standardize patterns (*zheng*).

### 2.4. Statistical Analysis

All data analyses were performed by RStudio (v4.0.3) software. The frequency, property, flavor, and meridian tropism of herbs were analyzed. *Arules* and *arulesViz* packages [8, 9] based on Apriori algorithm were used to analyze the association rules between herbs. The *flexclust*, *dendextend*, and *magrittr* packages [10, 11] were used to construct a hierarchical cluster analysis based on Euclidean metric, and a cluster dendrogram was drew. Both Apriori algorithm and hierarchical cluster have been successfully applied in data-mining analyses on Chinese medicine [12, 13].

## 3. Results

A total of 17,349 records were obtained, of which 8,414 were duplicate records and 7,990 were excluded according to the eligibility criteria. A total of 945 studies involved 970 Chinese prescriptions were included.

### 3.1. Overall Characteristics of Herbs in Prescriptions

#### 3.1.1. Frequency of Herbs in Prescriptions

The included 970 Chinese prescriptions involved 428 herbs, and 31 herbs were used more than 100 times as it is shown in Table 1. The most frequently prescribed herbs were Astragali Radix (Huangqi), which appeared 621 times, accounting for 64% of all herbs, followed by Atractylodis Macrocephalae Rhizome (Baizhu) (436 times, 45%), Glycyrrhizae Radix Rhizome (Gancao) (412 times, 42%), Poria (Fuling) (402 times, 42%), Ophiopogonis Radix (Maidong) (374 times, 39%), Hedyotidis Diffusae Herba (Baihuasheshecao) (306 times, 36%), and Codonopsis Radix (Dangshen) (299 times, 31%).

#### 3.1.2. Properties, Flavors, and Meridian Tropisms of Herbs

**Figure 1:**
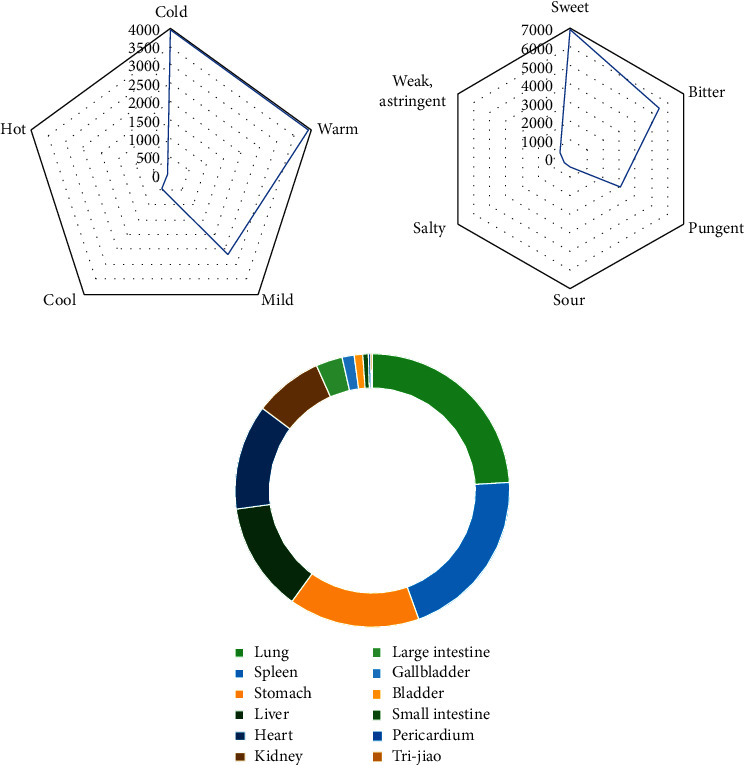
Property, flavor, and meridian tropism of herbs. (a) The radar chart of properties of herbs. (b) The radar chart of flavors of herbs. (c) The circle chart of meridian tropisms of herbs.

The most frequently appeared herbal property was cold, followed by warm, mild, and cool (Figure 1(a)). The flavors of herbs (Figure 1(b)) were mainly sweet, bitter, pungent, weak, astringent, sour, and salty. Herbs (Figure 1(c)) were mainly distributed to Lung Meridian of Hand-Taiyin, Spleen Meridian of Foot-Taiyin, Stomach Meridian of Foot-Yangming, Liver Meridian of Foot-Jueyin, Heart Meridian of Hand-Shaoyin, and Kidney Meridian of Foot-Shaoyin.

#### 3.1.3. Associations between Herbs

**Figure 2:**
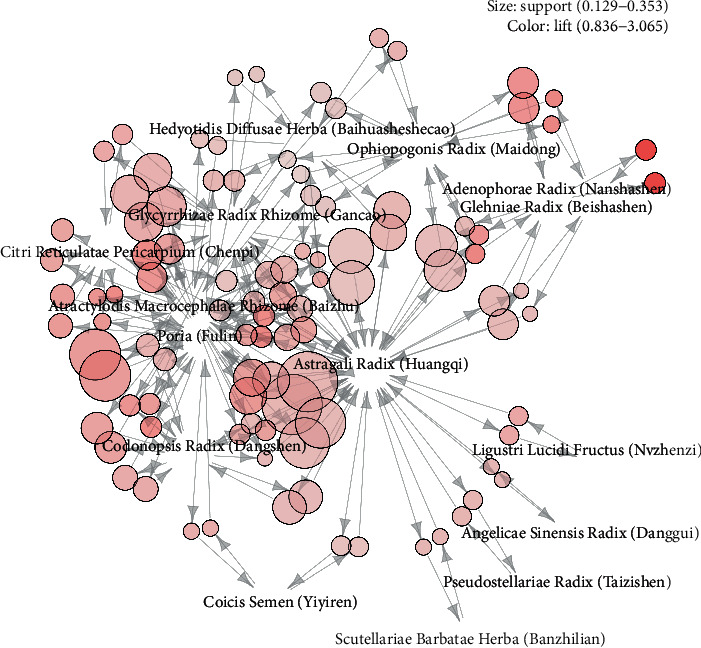
Association rule map of herbs based on graph method.

In association rule analysis, the minimum support level was set to 15% and the minimum confidence level was set to 60% in order to obtain frequently used herb pairs. A total of 25 rules were obtained, and the detailed information of them was listed in Table 2. Herb pairs with the highest degree of support was {Atractylodis Macrocephalae Rhizome (Baizhu)} => {Astragali Radix (Huangqi)}, followed by {Poria (Fuling)} => {Atractylodis Macrocephalae Rhizome (Baizhu)}, {Poria (Fuling)} => {Astragali Radix (Huangqi)}, {Glycyrrhizae Radix Rhizome (Gancao)} => {Astragali Radix (Huangqi)} and {Ophiopogonis Radix (Maidong)} => {Astragali Radix (Huangqi)}. Herb pairs with the highest confidence and the degree of lift were {Ligustri Lucidi Fructus (Nvzhenzi)} => {Astragali Radix (Huangqi)} and {Adenophorae Radix (Nanshashen)} => {Glehniae Radix (Beishashen)}.  Figure 2 visualizes the association rules by a graphics-based visualization technique. The depth of the color and the size of the vertex show the strength of association. Astragali Radix (Huangqi), Atractylodis Macrocephalae Rhizome (Baizhu), Poria (Fuling), and Glycyrrhizae Radix Rhizome (Gancao) were identified as hubs in association rules graph (Figure 2).

**Figure 3:**
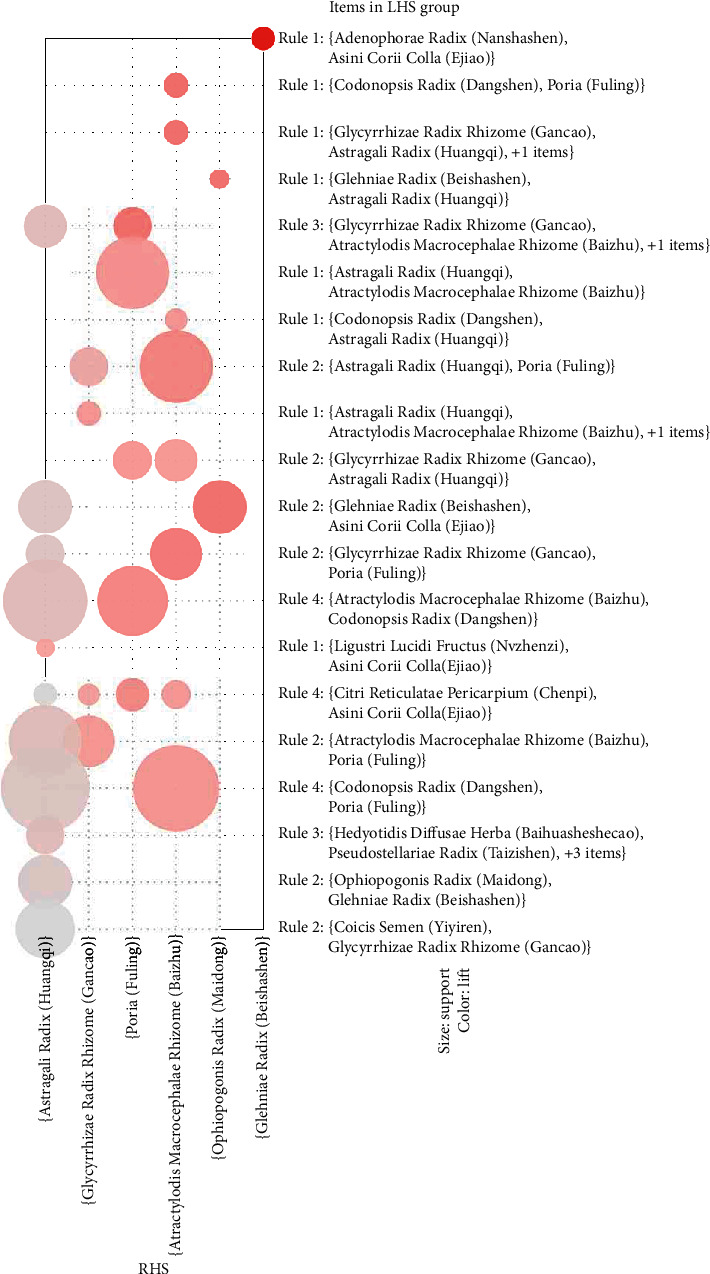
Association rule diagram of herbs based on grouped matrix method.

The general distribution of association rules is displayed in the grouping matrix diagram (Figure 3) which not only reveals the general rule but also extracts rules that shared commonalities. In the grouping matrix diagram, *X*-axis and *Y*-axis represent the right-hand side and left-hand side, respectively. The depth of a circle's color indicates the degree of lift; the darker the color, the higher the degree of lift. The size of a circle represents the degree of support. As the degree of support becomes higher, the circle becomes larger. Nine core herbs of paired prescriptions were identified: Atractylodis Macrocephalae Rhizome (Baizhu), Poria (Fuling), Citri Reticulatae Pericarpium (Chenpi), Glehniae Radix (Beishashen), Ophiopogonis Radix (Maidong), Codonopsis Radix (Dangshen), Glycyrrhizae Radix Rhizome (Gancao), Hedyotidis Diffusae Herba (Baihuasheshecao), and Astragali Radix (Huangqi).

#### 3.1.4. Cluster Analysis of Herbs

**Figure 4:**
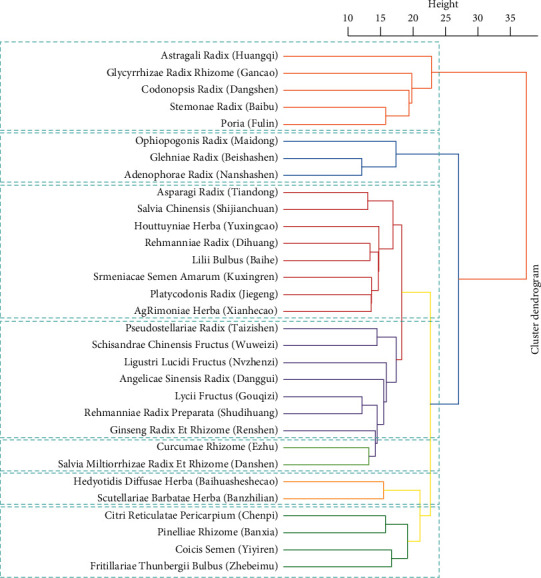
Cluster analysis of the 31 most frequently prescribed herbs.

A cluster analysis was performed based on herbs that appeared over 100 times, and 7 clusters were obtained (Figure 4). Cluster 1 included Astragali Radix (Huangqi), Glycyrrhizae Radix Rhizome (Gancao), Codonopsis Radix (Dangshen), Atractylodis Macrocephalae Rhizome (Baizhu), and Poria (Fuling). Cluster 2 contained Ophiopogonis Radix (Maidong), Glehniae Radix (Beishashen) and Adenophorae Radix (Nanshashen). Cluster 3 consisted of Asparagi Radix (Tiandong), Salvia Chinensis (Shijianchuan), Houttuyniae Herba (Yuxingcao), Rehmanniae Radix (Dihuang), Lilii Bulbus (Baihe), Armeniacae Semen Amarum (Kuxinren), Platycodonis Radix (Jiegeng), and Agrimoniae Herba (Xianhecao). Cluster 4 contained Pseudostellariae Radix (Taizishen), Schisandrae Chinensis Fructus, Ligustri Lucidi Fructus (Nvzhenzi), Sinensis Radix (Danggui), Lycii Fructus (Gouqizi), Rehmanniae Radix Preparata (Shudihuang), and Ginseng Radix Et Rhizome (Renshen). Cluster 5 was formed by Curcumae Rhizome (Ezhu), and Salvia Miltiorrhizae Radix Et Rhizome (Danshen). Cluster 6 involved Hedyotidis Diffusae Herba (Baihuasheshecao) and Scutellariae Barbatae Herba (Banzhilian). Cluster 7 included Citri Reticulatae Pericarpium (Chenpi), Pinelliae Rhizome (Banxia), Coicis Semen (Yiyiren), and Fritillariae Thunbergii Bulbus (Zhebeimu).

### 3.2. Prescription Patterns of Herbs

Seven patterns (*zheng*) were identified from 970 Chinese prescriptions, including 705 prescriptions related to *qi* deficiency pattern, 364 prescriptions related to *yin* deficiency pattern, 355 prescriptions related to heat toxin pattern, 349 prescriptions related to phlegm-dampness pattern, 312 prescriptions related to kidney deficiency pattern, 219 prescriptions related to blood deficiency pattern, and 145 prescriptions related to blood stasis pattern.

**Figure 5:**
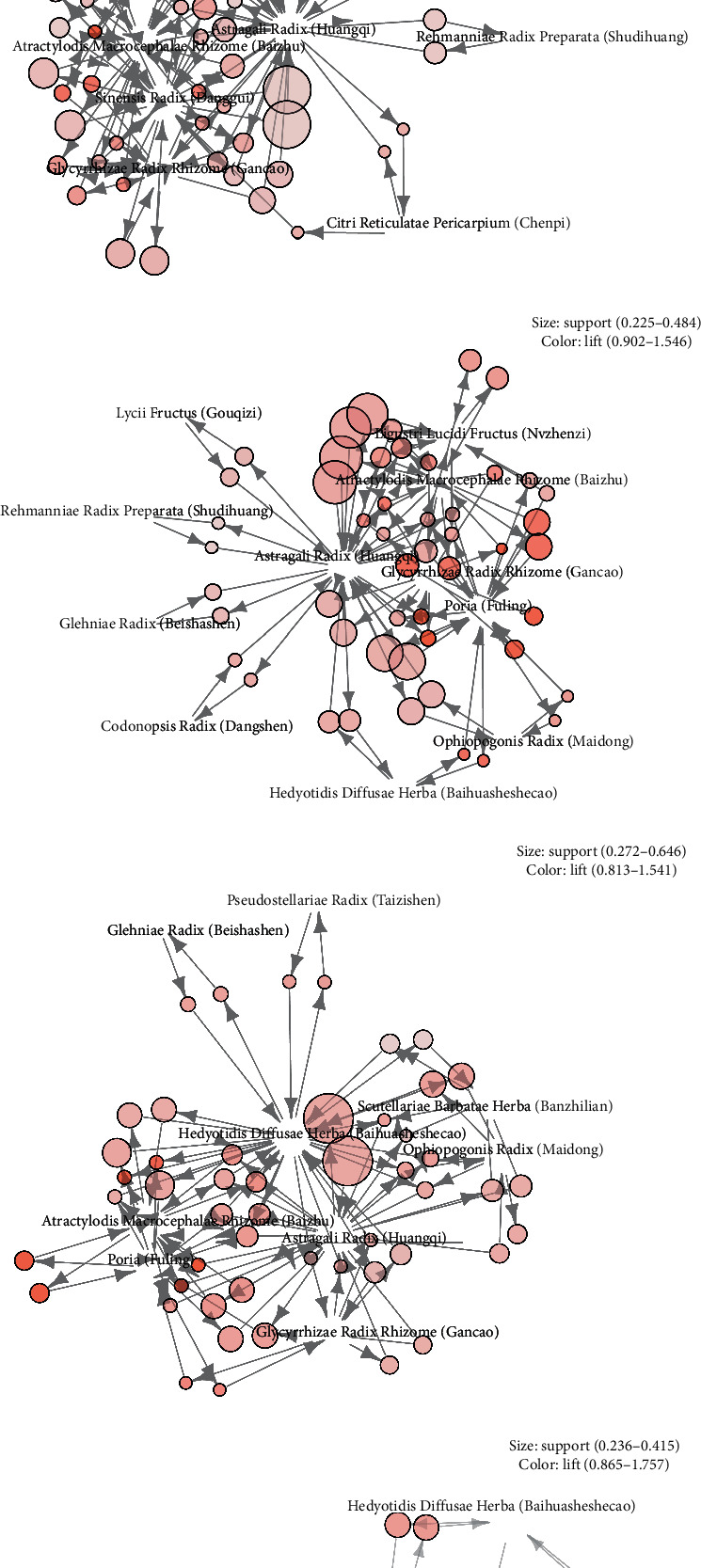
Association rule combination matrix of different patterns (*zheng*). (a) Association rule diagram of qi deficiency pattern. (b) Association rule diagram of yin deficiency pattern. (c) Association rule diagram of blood deficiency pattern. (d) Association rule diagram of kidney deficiency pattern. (e) Association rule diagram of heat toxin pattern. (f) Association rule diagram of phlegm-dampness pattern. (g) Association rule diagram of blood stasis pattern.

The top 10 most frequently used herbs in treating different patterns are presented in Table 3 and their association rule diagrams are shown in Figure 5. Astragali Radix (Huangqi) was the most frequently used herb in treating *qi* deficiency pattern, *yin* deficiency pattern, blood deficiency pattern, kidney deficiency pattern, and blood stasis pattern, while Hedyotidis Diffusae Herba (Baihuasheshecao) was the most frequently prescribed herb in treating heat toxin pattern and Coicis Semen (Yiyiren) in treating phlegm-dampness pattern. Most of high-frequency herbs on each pattern were also herbs with effects that are in accordance with the pattern. For example, Astragali Radix (Huangqi), Atractylodis Macrocephalae Rhizome (Baizhu) and Codonopsis Radix (Dangshen) on qi deficiency pattern, Ophiopogonis Radix (Maidong), Tiandong, Glehniae Radix (Beishashen) on yin deficiency pattern, Sinensis Radix (Danggui) and Rehmanniae Radix Preparata (Shudihuang) on blood deficiency pattern, Ligustri Lucidi Fructus (Nvzhenzi) and Lycii Fructus (Gouqizi) on kidney deficiency pattern, Scutellariae Barbatae Herba (Banzhilian) and Hedyotidis Diffusae Herba (Baihuasheshecao) on heat toxin pattern, Fritillariae Thunbergii Bulbus (Zhebeimu) and Poria (Fuling) on phlegm-dampness pattern, Curcumae Rhizome (Ezhu) and Salvia Miltiorrhizae Radix Et Rhizome (Danshen) on blood stasis pattern.

Paired herbs and herb groups on different patterns were extracted by association rules analysis (Figure 5). Astragali Radix (Huangqi)-Atractylodis Macrocephalae Rhizome (Baizhu) was the most commonly used herb pair on *qi* deficiency pattern. Ophiopogonis Radix (Maidong), Glehniae Radix (Beishashen), and Adenophorae Radix (Nanshashen) formed a frequently used herb groups on *yin* deficiency pattern. In the treatment of blood deficiency pattern, Astragali Radix (Huangqi)-Angelicae Sinensis Radix (Danggui) was the most frequently prescribed herb pair. In treating kidney deficiency pattern, Astragali Radix (Huangqi) and Atractylodis Macrocephalae Rhizome (Baizhu) were located centrally in the graph and were prescribed with Ligustri Lucidi Fructus (Nvzhenzi), Lycii Fructus (Gouqizi), and Rehmanniae Radix Preparata (Shudihuang). In treating heat toxin pattern, Hedyotidis Diffusae Herba (Baihuasheshecao) and Scutellariae Barbatae Herba (Banzhilian) combined a pair of herbs. Poria (Fuling), Coicis Semen (Yiyiren), and Fritillariae Thunbergii Bulbus (Zhebeimu) formed an herb group on phlegm-dampness pattern. In treating blood stasis pattern, two herb groups were frequently used: Chuanxiong Rhizoma (Chuangxiong), Persicae Semen (Taoren), and Carthami Flos (Honghua); Curcumae Rhizome (Ezhu) and Sparganii Rhizoma (Sanleng).

## 4. Discussion

### 4.1. To Clear and Tonify Lung-Spleen Is the Principal Therapy of TCM on NSCLC

In clinical practice, the properties and actions of herbs are essential bases for practitioners to apply herbal medicines based on therapeutic principles of TCM, theories of *yin-yang* and *zang-fu*, and meridian tropisms [14]. In this study, the ten most frequently used herbs and the five most commonly used herb pairs all targeted the spleen and stomach, while the herbs on NSCLC are mainly distributed to lung, spleen, and stomach channels. It indicated that lung and spleen were key viscera in the treatment of NSCLC. The properties of the herbs were mainly sweet and cold, sweet and warm, bitter and cold, and bitter and warm, which were commonly used in clearing method and tonifying method. Thus, the findings suggested that applying clearing and tonifying method by targeting the lung and spleen was the most widely used therapy in the treatment of NSCLC. TCM theories provide explanations for the findings in two aspects. On the one hand, the spleen is considered as the source of the production and transformation of *qi* and blood. Chinese medicine practitioners attach importance to strengthening and harmonizing the spleen in the treatment of chronic diseases. On the other hand, the spleen is the source of phlegm while the lung is the storage vessel. Therefore, fortifying the spleen to reduce phlegm and alleviate symptoms of the lung has become an important method of treatment in patients with NSCLC.

### 4.2. Potential Effective Medicinal Compatibility of TCM on NSCLC

The cluster analysis divided the herbs into seven clusters (Figure 4). Herbs in cluster 1 were among the most frequently used herbs and prescribed to fortify the spleen, replenish *qi*, and drain dampness. Herbs in cluster 2 were prescribed to nourish the *yin* of the lung and stomach. Herbs in cluster 3 were mainly used to nourish *yin*, clear heat, diffuse the lung, and resolve phlegm. Herbs in cluster 4 were mainly prescribed to nourish the *yin* of the kidney and lung and replenish *qi*. Cluster 5 consisted of herbs for activating blood and resolving stasis, while cluster 6 included heat-clearing and detoxicating herbs. Cluster 7 contained herbs for regulating *qi*, draining dampness, resolving phlegm, and dissipating binds. These clusters provided an available reference for medicinal compatibility in the treatment of NSCLC.

### 4.3. Pattern (*Zheng*) Differentiation and Treatment of TCM on NSCLC

Visualized by modern computer technology, the high-frequency herbs, herb pairs, and herb groups on different patterns offered a glimpse of unique views of TCM on the cause, mechanism, and treatment of NSCLC. (1) The high-frequency herbs on *qi* deficiency pattern were mainly distributed to the lung and spleen, indicating that dual deficiency of the lung-spleen was common in NSCLC patients with *qi* deficiency pattern. High-frequency herbs on *yin* deficiency pattern were mainly distributed to the lung and stomach, suggesting that *yin* deficiency patterns of NSCLC were mainly manifested in the lung and stomach.

1. High-frequency herbs on blood deficiency pattern generally targeted the spleen and stomach, which reflected that the spleen and stomach were regarded as sources of *qi* and blood in TCM. As patients with blood deficiency are prone to blood stasis, Astragali Radix (Huangqi) was often prescribed with Sinensis Radix (Danggui) (Figure 5(c)) to fortify the spleen and replenish *qi* and tonify and activate blood as well.
2. High-frequency herbs on kidney deficiency pattern included more herbs for tonifying the spleen than herbs for tonifying the kidney, and no herb for tonifying the kidney *yang* was included, indicating that tonifying the spleen and the kidney *yin* was a major method in treating kidney deficiency pattern of NSCLC. Herb groups on kidney deficiency pattern also supported the findings (Figure 5(d)).
3. In the treatment of heat toxin pattern, two heat-clearing and detoxicating herbs, Hedyotidis Diffusae Herba (Baihuasheshecao) and Scutellariae Barbatae Herba (Banzhilian), formed an herb pair (Figure 5(e)). Hedyotidis Diffusae Herba (Baihuasheshecao) was the only heat-clearing and detoxicating herb that was listed in the ten most frequently prescribed herbs. It was also one of the high-frequency herbs on the common patterns except blood deficiency. It is reported that chemical constituents of Hedyotidis Diffusae Herba (Baihuasheshecao) exhibited cytotoxicity to tumor cell lines [15] and suppressed proliferation of various types of tumor cells often through inducting apoptosis or affecting cell cycle progression [16]. Hedyotidis Diffusae Herba (Baihuasheshecao) is a source of anticancer agents warranting further study.
4. Herbs with corresponding effects were identified as herb groups or herb pairs on phlegm-dampness pattern and blood stasis pattern (Figures 5(f) and 5(g)). In treating these patterns, herbs for tonifying the lung and spleen were prescribed with herbs for reducing phlegm-dampness and blood stasis, suggesting that deficiency in the root caused the accumulation of pathological products and resulted in excess syndromes.
5. Herbs for fortifying the spleen, tonifying the lung, and replenishing *qi* were identified as high-frequency herbs in seven patterns, which added to the support for the method of clearing and tonifying the lung and spleen in the treatment of NSCLC.

## 5. Conclusion

Our study found that applying clearing and tonifying method through targeting the lung and spleen was the most widely used therapy in treating NSCLC. The ten most frequently used herbs were identified. The 428 herbs were generally distributed to lung, spleen, stomach, liver, and heart channels. Atractylodis Macrocephalae Rhizome (Baizhu) paired with Astragali Radix (Huangqi), Poria (Fuling) paired with Atractylodis Macrocephalae Rhizome (Baizhu), Poria (Fuling) paired with Astragali Radix (Huangqi), Glycyrrhizae Radix Rhizome (Gancao) paired with Astragali Radix (Huangqi), and Ophiopogonis Radix (Maidong) paired with Astragali Radix (Huangqi) were the five most frequently used herb pairs. *Qi* deficiency pattern, *yin* deficiency pattern, blood deficiency pattern, kidney deficiency pattern, heat toxin pattern, phlegm-dampness pattern, and blood stasis pattern were the most common syndromes in NSCLC.

## Figures and Tables

**Table 1:** Herbs appearing over 100 times in prescriptions.

Herb	Frequency	Rate	Herb	Frequency	Rate
Astragali Radix (Huangqi)	621	0.64	Ligustri Lucidi Fructus (Nvzhenzi)	157	0.16
Atractylodis Macrocephalae Rhizome (Baizhu)	436	0.45	Asparagi Radix (Tiandong)	145	0.15
Glycyrrhizae Radix Rhizome (Gancao)	412	0.42	Platycodonis Radix (Jiegeng)	135	0.14
Poria (Fuling)	405	0.42	Schisandrae Chinensis Fructus (Wuweizi)	127	0.13
Ophiopogonis Radix (Maidong)	374	0.39	Rehmanniae Radix (Dihuang)	127	0.13
Hedyotidis Diffusae Herba (Baihuasheshecao)	306	0.32	Houttuyniae Herba (Yuxingcao)	126	0.13
Codonopsis Radix (Dangshen)	299	0.31	Armeniacae Semen Amarum (Kuxinren)	125	0.13
Glehniae Radix (Beishashen)	278	0.29	Ginseng Radix Et Rhizome (Renshen)	121	0.12
Citri Reticulatae Pericarpium (Chenpi)	238	0.25	Lilii Bulbus (Baihe)	119	0.12
Coicis Semen (Yiyiren)	230	0.24	Curcumae Rhizome (Ezhu)	118	0.12
Fritillariae Thunbergii Bulbus (Zhebeimu)	212	0.22	Salvia Chinensis (Shijianchuan)	110	0.11
Pinelliae Rhizome (Banxia)	202	0.21	Agrimoniae Herba (Xianhecao)	104	0.11
Pseudostellariae Radix (Taizishen)	191	0.20	Salvia Miltiorrhizae Radix Et Rhizome (Danshen)	102	0.11
Adenophorae Radix (Nanshashen)	178	0.18	Lycii Fructus (Gouqizi)	102	0.11
Scutellariae Barbatae Herba (Banzhilian)	174	0.18	Rehmanniae Radix Preparata (Shudihuang)	101	0.10
Angelicae Sinensis Radix (Danggui)	173	0.18			

**Table 2:** Association rules of herbs on NSCLC.

Items (left-hand side => right-hand side)	Support	Confidence	Lift
{Atractylodis Macrocephalae Rhizome (Baizhu)} => {Astragali Radix (Huangqi)}	0.35	0.80	1.26
{Poria (Fuling)} => {Atractylodis Macrocephalae Rhizome (Baizhu)}	0.31	0.74	1.66
{Poria (Fuling)} => {Astragali Radix (Huangqi)}	0.30	0.73	1.15
{Glycyrrhizae Radix Rhizome (Gancao)} => {Astragali Radix (Huangqi)}	0.28	0.65	1.03
{Ophiopogonis Radix (Maidong)} => {Astragali Radix (Huangqi)}	0.26	0.69	1.10
{Poria (Fuling), Astragali Radix (Huangqi)} => {Atractylodis Macrocephalae Rhizome (Baizhu)}	0.24	0.78	1.76
{Hedyotidis Diffusae Herba (Baihuasheshecao)} => {Astragali Radix (Huangqi)}	0.24	0.77	1.21
{Codonopsis Radix (Dangshen => {Astragali Radix (Huangqi)}	0.22	0.72	1.14
{Codonopsis Radix (Dangshen)} => {Atractylodis Macrocephalae Rhizome (Baizhu)}	0.21	0.69	1.55
{Glehniae Radix (Beishashen)} => {Ophiopogonis Radix (Maidong)}	0.21	0.73	1.93
{Atractylodis Macrocephalae Rhizome (Baizhu), Glycyrrhizae Radix Rhizome (Gancao)} => {Poria (Fuling)}	0.20	0.83	1.99
{Atractylodis Macrocephalae Rhizome (Baizhu), Glycyrrhizae Radix Rhizome (Gancao)} => {Astragali Radix (Huangqi)}	0.19	0.77	1.22
{Poria (Fuling), Glycyrrhizae Radix Rhizome (Gancao)} => {Astragali Radix (Huangqi)}	0.18	0.73	1.16
{Citri Reticulatae Pericarpium (Chenpi)} => {Poria (Fuling)}	0.17	0.71	1.70
{Citri Reticulatae Pericarpium (Chenpi)} => {Atractylodis Macrocephalae Rhizome (Baizhu)}	0.17	0.68	1.53
{Codonopsis Radix (Dangshen), Poria (Fuling)} => {Atractylodis Macrocephalae Rhizome (Baizhu)}	0.16	0.89	2.01
{Poria (Fuling), Glycyrrhizae Radix Rhizome (Gancao), Astragali Radix (Huangqi)} => {Atractylodis Macrocephalae Rhizome (Baizhu)}	0.16	0.87	1.97
{Citri Reticulatae Pericarpium (Chenpi)} => {Astragali Radix (Huangqi)}	0.16	0.65	1.02
{Adenophorae Radix (Nanshashen)} => {Glehniae Radix (Beishashen)}	0.16	0.87	3.07
{Atractylodis Macrocephalae Rhizome (Baizhu), Codonopsis Radix (Dangshen)} => {Astragali Radix (Huangqi)}	0.16	0.74	1.17
{Citri Reticulatae Pericarpium (Chenpi)} => {Glycyrrhizae Radix Rhizome (Gancao)}	0.16	0.63	1.49
{Coicis Semen (Yiyiren)} => {Astragali Radix (Huangqi)}	0.15	0.64	1.02
{Glehniae Radix (Beishashen), Astragali Radix (Huangqi)} => {Ophiopogonis Radix (Maidong)}	0.15	0.73	1.94
{Ligustri Lucidi Fructus (Nvzhenzi)} => {Astragali Radix (Huangqi)}	0.15	0.93	1.47
{Pseudostellariae Radix (Taizishen)} => {Astragali Radix (Huangqi)}	0.15	0.77	1.22

**Table 3:** The top 10 herbs on different patterns (*zheng*) of NSCLC.

High-frequency herbs on *qi* deficiency pattern	Frequency	High-frequency herbs on *yin* deficiency pattern	Frequency
Astragali Radix (Huangqi)	619	Astragali Radix (Huangqi)	265
Atractylodis Macrocephalae Rhizome (Baizhu)	433	Ophiopogonis Radix (Maidong)	261
Poria (Fuling)	363	Glehniae Radix (Beishashen)	196
Glycyrrhizae Radix Rhizome (Gancao)	320	Atractylodis Macrocephalae Rhizome (Baizhu)	139
Ophiopogonis Radix (Maidong)	271	Glycyrrhizae Radix Rhizome (Gancao)	133
Codonopsis Radix (Dangshen)	268	Poria (Fuling)	133
Hedyotidis Diffusae Herba (Baihuasheshecao)	249	Hedyotidis Diffusae Herba (Baihuasheshecao)	129
Glehniae Radix (Beishashen)	210	Adenophorae Radix (Nanshashen)	124
Citri Reticulatae Pericarpium (Chenpi)	195	Asparagi Radix (Tiandong)	100
Coicis Semen (Yiyiren)	175	Pseudostellariae Radix (Taizishen)	99
High frequency herbs on blood deficiency pattern	Frequency	High-frequency herbs on kidney deficiency pattern	Frequency
Astragali Radix (Huangqi)	170	Astragali Radix (Huangqi)	264
Sinensis Radix (Danggui)	168	Atractylodis Macrocephalae Rhizome (Baizhu)	163
Atractylodis Macrocephalae Rhizome (Baizhu)	114	Ligustri Lucidi Fructus (Nvzhenzi)	157
Glycyrrhizae Radix Rhizome (Gancao)	104	Poria (Fuling)	151
Rehmanniae Radix Preparata (Shudihuang)	98	Ophiopogonis Radix (Maidong)	126
Poria (Fuling)	94	Glycyrrhizae Radix Rhizome (Gancao)	125
Ophiopogonis Radix (Maidong)	72	Hedyotidis Diffusae Herba (Baihuasheshecao)	110
Codonopsis Radix (Dangshen)	65	Lycii Fructus (Gouqizi)	102
Citri Reticulatae Pericarpium (Chenpi)	63	Glehniae Radix (Beishashen)	100
Pseudostellariae Radix (Taizishen)	57	Rehmanniae Radix Preparata (Shudihuang)	98
High frequency herbs on heat toxin pattern	Frequency	High frequency herbs on phlegm-dampness pattern	Frequency
Hedyotidis Diffusae Herba (Baihuasheshecao)	302	Coicis Semen (Yiyiren)	227
Astragali Radix (Huangqi)	266	Astragali Radix (Huangqi)	220
Poria (Fuling)	174	Fritillariae Thunbergii Bulbus (Zhebeimu)	211
Scutellariae Barbatae Herba (Banzhilian)	173	Poria (Fuling)	176
Atractylodis Macrocephalae Rhizome (Baizhu)	164	Atractylodis Macrocephalae Rhizome (Baizhu)	163
Ophiopogonis Radix (Maidong)	161	Glycyrrhizae Radix Rhizome (Gancao)	161
Glycyrrhizae Radix Rhizome (Gancao)	157	Ophiopogonis Radix (Maidong)	135
Glehniae Radix (Beishashen)	116	Hedyotidis Diffusae Herba (Baihuasheshecao)	125
Pseudostellariae Radix (Taizishen)	109	Codonopsis Radix (Dangshen)	118
Coicis Semen (Yiyiren)	106	Glehniae Radix (Beishashen)	109
High frequency herbs on blood stasis pattern	Frequency		
Astragali Radix (Huangqi)	92				
Glycyrrhizae Radix Rhizome (Gancao)	63				
Poria (Fuling)	60				
Atractylodis Macrocephalae Rhizome (Baizhu)	55				
Codonopsis Radix (Dangshen)	48				
Hedyotidis Diffusae Herba (Baihuasheshecao)	48				
Curcumae Rhizome (Ezhu)	47				
Salvia Miltiorrhizae Radix Et Rhizome (Danshen)	41				
Persicae Semen (Taoren)	38				
Sinensis Radix (Danggui)	37				

## Data Availability

The data used to support the findings of this study are included within the article.
